# Characteristics of Drug-Susceptible and Drug-Resistant *Staphylococcus aureus* Pneumonia in Patients with HIV

**DOI:** 10.4172/2161-1165.1000122

**Published:** 2013-05-06

**Authors:** Charles K. Everett, Anuradha Subramanian, Leah G. Jarisberg, Matthew Fei, Laurence Huang

**Affiliations:** 1 Division of Pulmonary and Critical Care Medicine, University of California, San Francisco, San Francisco General Hospital, 1001 Potrero Avenue, Box 0841, San Francisco, CA, USA; 2 Division of HIV/AIDS, University of California, San Francisco, San Francisco General Hospital, Ward 84, 995 Potrero Avenue, San Francisco, CA 94110, USA; 3 Division of Infectious Diseases, Department of Medicine, University of Maryland, 655 West Baltimore Street, Baltimore, MD 21201, USA; 4 Division of Pulmonary and Critical Care Medicine, Department of Medicine, Group Health Bellevue Medical Center, 11511 NE 10th St # 3, Bellevue, WA, USA

**Keywords:** HIV, AIDS, Pneumonia, Staphylococcus aureus, Staphylococcal pneumonia, MRSA Streptococcus pneumonia, Pneumococcal pneumonia

## Abstract

**Objectives:**

To examine predictors and outcomes of *Staphylococcus aureus* Pneumonia (SAP) in people with HIV compared with *Streptococcus pneumoniae* Pneumonia (SPP), and to compare Methicillin-Resistant *S. aureus* (MRSA) with Methicillin-Sensitive *S. aureus* (MSSA) pneumonias in this population.

**Methods:**

We conducted a retrospective case-control study of HIV-infected patients admitted to a single center with culture-proven *S. aureus* or *S. pneumoniae* pneumonia. We identified patients through a computerized database, conducted structured chart reviews, and performed bivariate and multivariate analyses using logistic regression.

**Results:**

We compared 47 SAP episodes in 42 patients with 100 SPP episodes in 93 patients. Use of any antibiotics prior to admission (OR=3.5, p=0.02), a co-morbid illness (OR=4.2, p=0.04), and recent healthcare contact (OR=12.0, p<0.001) were significant independent predictors of SAP. Patients with SAP were more likely to require intensive care (OR=2.7, p=0.02) and mechanical ventilation (OR=3.1, p=0.02), but not to die. MRSA was more common (57% of cases) than MSSA, but outcomes were not significantly worse.

**Conclusions:**

Patients with HIV and SAP have worse outcomes than those with SPP. Clinicians should consider empiric antibiotic coverage for MRSA in patients admitted with HIV and pneumonia, given the high prevalence of MRSA. Further studies are warranted to examine morbidity differences between HIV-associated MSSA and MRSA pneumonia.

## Introduction

Bacterial pneumonia is a well-recognized complication of HIV infection. The risk for HIV-associated bacterial pneumonia increases with declining CD_4_ cell counts and persists despite decreased opportunistic complications and improved survival due to Antiretroviral Therapy (ART) [1, 2]. Despite widespread access to ART, bacterial Community-Acquired Pneumonia (CAP) remains the most common pulmonary complication of HIV disease, with a 5-fold greater incidence in people with HIV [3]. Mortality may be increasing due to the prevalence of co-morbid conditions, in particular hepatic cirrhosis, among patients hospitalized with CAP [4–7]. *Streptococcus pneumoniae* is the most commonly isolated pathogen in patients with HIV and CAP [1, 8, 9]. Many of the studies that established the risk factors for CAP and its outcomes in people with HIV focused specifically on invasive pneumococcal disease, while others did not clearly establish microbiological etiology but must have included large numbers of patients with pneumococcus [7–10].

In contrast, relatively little is known about the role that other important pathogens, including *Staphylococcus aureus*, play in HIV-associated CAP. Studies have suggested that *S. aureus* causes roughly 15% of microbiologically confirmed CAP cases in people with HIV [1, 11]. Despite its frequency, few studies have closely examined *S. aureus* Pneumonia (SAP) in patients with HIV. Early in the HIV epidemic, *S. aureus* was commonly cultured from sputum [12] and SAP was associated with Injection Drug Use (IDU), previous PCP, and cirrhosis [13], but these studies did not identify risk factors for SAP in comparison to other causes of CAP. In the intervening period,

Complicated and severe infections due to *S. aureus*, in particular Methicillin-Resistant *S. aureus* (MRSA) associated with community-acquired drug resistance (USA300 family), have increased markedly in people with HIV [14] and in the community [15, 16].

To better characterize CAP due to *S. aureus* in patients with HIV, we reviewed all cases of SAP over a 13-year period at our institution. Our goals were to characterize the risk factors for SAP generally and MRSA specifically, to compare the outcomes for SAP with those for *S. pneumoniae* Pneumonia (SPP), and those for Methicillin-Sensitive *S. aureus* (MSSA) pneumonia with MRSA.

## Patients and Methods

### Study design and subjects

We conducted a case-control study within a retrospective cohort of HIV-infected adults admitted with pneumonia to San Francisco General Hospital (SFGH) between June 1996 and June 2008. We identified subjects with a computerized medical records search using the International Classification of Diseases, ninth revision (ICD-9), codes for HIV (042), *S. pneumoniae* pneumonia (481, or 482.3), and *S. aureus* pneumonia (482.4, 482.40, 482.41, or 482.49). *S. aureus* cases were HIV-infected patients identified by ICD-9 code for *S. aureus* pneumonia who also met the study definition of SAP. After determining the total number of potential *S. pneumoniae* control cases and targeting approximately 2 controls for each case, every fourth chart identified by the ICD-9 search for *S. pneumoniae* pneumonia was reviewed and the first 100 cases meeting the study definition of SPP were included as controls. We excluded cases meeting the definition of hospital-acquired pneumonia established by the Infectious Disease Society of America/American Thoracic Society 2005 guidelines [17]. The University of California, San Francisco Committee on Human Research approved the study protocol (IRB approval number 10-02623)

### Case definitions

The following criteria were used to diagnose SAP: (1) culture from blood, pleural fluid, or BAL showing *S. aureus*, an abnormal chest radiograph, and clinical evidence of pneumonia; or (2) clinical and radiographic findings consistent with pneumonia, *S. aureus* from sputum, and therapy directed against *S. aureus*. SPP was diagnosed similarly except that empiric therapy for CAP was considered specific for *S. pneumoniae*.

Microbiological isolates of *S. aureus* were defined as methicillin-resistant based on antibiotic sensitivity testing performed according to standards set out by the Clinical Laboratory Standards Institute [18]. Briefly, *S. aureus* isolates were placed in oxacillin and cefoxitin wells to determine the Minimal Inhibitory Concentrations (MIC) for each drug. A cefoxitin disk diffusion test was performed to confirm MIC results. Isolates were defined as “methicillin resistant” if both MIC and disk diffusion showed resistance according to the following cut points: oxacillin MIC ≥ 4 micrograms/ml and cefoxitin MIC ≥ 8 micrograms/ml on the MicroScan tray; cefoxitin disk diffusion ≤ 21 mm.

### Data collection

Investigators (C.K.E., A.S., and M.F.) reviewed the medical records using standardized data extraction forms. Recent healthcare contact was defined as any hospital admission, residence in a nursing home or skilled nursing facility, or dialysis in the two months prior to admission. Clinical information included patient demographics; housing status; current alcohol, drug and tobacco use; presence of co-morbid diagnoses; prior pneumococcal vaccination; current use of antiretroviral therapy and opportunistic infection prophylaxis; vital signs on admission; CD_4_ count and plasma HIV RNA level within one month prior to or immediately upon admission; basic labs at admission; initial antibiotic therapy; and the need for chest tube placement, ICU transfer, and mechanical ventilation. The primary outcome was survival to hospital discharge. Secondary outcomes were survival at 6 weeks following discharge and the requirement for ICU level care, mechanical ventilation, or chest tube. The data were entered into a customized database (Access 2003, Microsoft, Redmond, Washington) and were subjected to electronic validation rules.

### Statistical analysis

Bivariate associations were tested using the Chi squared test for categorical variables and Student’s t-test for continuous variables. Unadjusted odds ratios were calculated using bivariate logistic regression. Adjusted odds were calculated using multivariate logistic regression with SAP or MRSA as the dependent variable and included all predictors that had p <0.20 in bivariate analysis. When performing logistic regression, general estimating equation methodology was used to account for pneumonia case clustering by patient. Significance was defined as having p<0.05. All statistics were calculated using SAS 9.2 (SAS Institute Inc., Cary, NC).

## Results

**Figure 1:**
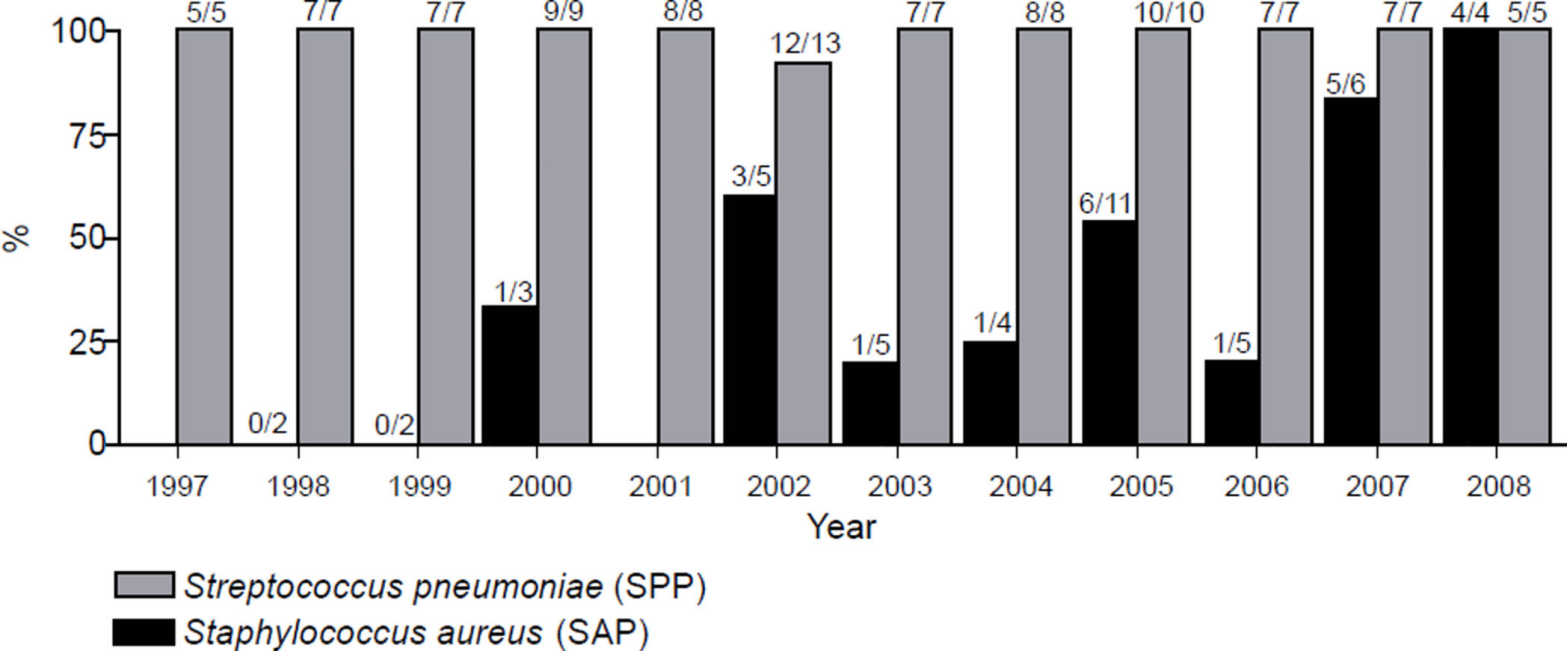
Percent and number of patients with *S. pneumoniae* (SPP) or *S. aureus* pneumonia (SAP) who received appropriate antibiotic therapy on the first day of treatment by year of hospital admission. Note: SPP= *S. pneumoniae* pneumonia; SAP= *S. aureus* pneumonia; SAP cases total 47, SPP cases total 93

Fifty-two episodes of *S. aureus* pneumonia occurred among 47 HIV-infected patients during the study period. Five hospital-acquired cases of SAP were excluded subsequently, leaving 47 SAP cases among 42 patients. These were compared to 100 SPP cases from 93 patients. The number of SAP cases each year fluctuated, with 11 cases in 2005 being the largest number for any year, while SPP cases were evenly distributed throughout the study period. Nearly all cases of SPP (92 of 93, 99%, antibiotic data was unavailable for 7 cases) were treated with at least one antibiotic directed at *S. pneumoniae* at the time of admission (Figure 1). In contrast, only in 2005, 2007, and 2008 did more than 50% of SAP cases receive an antibiotic directed against *S. aureus* on the day of admission.

Among SAP cases, 23 of 47 (49%) had positive blood cultures, 2 of the remaining 24 cases (4%) had positive pleural fluid cultures, and the other 22 (47%) had positive cultures from sputum, tracheal aspirate, or BAL fluid. Overall, 6 (13%) SAP cases had positive pleural fluid and 2 (4%) had positive BAL fluid. Among SPP cases, positive blood cultures were also common (74 of 100, 74%). All the remaining cases had positive sputum cultures, while no positive cultures were obtained from tracheal aspirate, pleural fluid, or BAL.

### Study population

The baseline characteristics were similar in patients with SAP and SPP, with a few notable differences (Table 1). Whites (33% of patients with SPP vs. 52% in patients with SAP, p=0.04) were less common and active smokers (80% vs. 50%, p<0.001, SPP vs. SAP respectively) more common among SPP cases. Any co-morbid illness (88% of patients with SAP versus 65% with SPP, p=0.004) and chronic lung disease (48% of patients with SAP vs. 30% with SPP, p<0.05) were significantly more common in SAP. Significantly more patients with SAP were taking PCP prophylaxis (55% for SAP vs. 22% for SPP, p<0.001) and specifically taking trimethoprim- sulfamethoxazole, TMP-SMX (33% for SAP vs. 15% for SPP, p=0.02). Roughly equal proportions of patients had been vaccinated against pneumococcus.

### Risk factors for *S. aureus* pneumonia

Co-morbid illness and increased engagement with the healthcare system seemed to increase the risk for SAP (Table 2) in multivariable adjusted analyses. Any antibiotic taken prior to admission (OR 3.5, 95% CI 1.3–9.9, p=0.02), co-morbid diagnosis (OR 4.2, 95% CI 1.1–16.6, p=0.04), and recent healthcare contact (OR 12.0, 95% CI 3.8–38.3, p<0.001) all resulted in strongly increased odds of SAP. The category “any antibiotic” includes both PCP prophylaxis and non-PCP prophylaxis (e.g., azithromycin for disseminated *Mycobacterium avium intracellulare* complex (MAC)), both of which conferred increased odds for SAP in unadjusted analyses but not after adjustment, suggesting that those variables define a single population of patients.

### Outcomes for *S. aureus* pneumonia

Diagnosis with SAP did not confer a greater risk of death in our cohort (Table 3). There were few deaths overall; 2 deaths in each group occurred prior to hospital discharge, with one additional death less than 6 weeks after discharge in a patient with SAP. However, a SAP diagnosis was associated with increased in-hospital morbidity. Patients with SAP were more likely to be admitted to the ICU (OR 2.7, 95% CI 1.2–6.1, p=0.02) and more likely to be incubated (OR 3.1, 95% CI 1.2–7.9, p=0.02). Chest tube placement also occurred more frequently in patients with SAP (OR 2.7, 95% CI 0.9–8.2, p=0.07), though the difference was not statistically significant. SAP patients had a longer median length of hospital stay (OR 1.08 per additional day, 95% CI 1.02–1.1, p=0.01). Finally, the odds of hospital readmission within 2 months were higher in SAP, with 49% of patients with SAP requiring readmission compared to 21% among those with SPP (OR 3.5, 95% CI 1.6–7.5, p<0.001).

### Risk factors and Outcomes for drug-resistant *S. aureus*

Among the 47 episodes of *S. aureus* pneumonia identified by this study, 46 had valid drug resistance data available and a total of 26 (57%) were documented by culture and sensitivity testing to be MRSA. PCP prophylaxis with TMP-SMX decreased the odds of MRSA (OR 0.2, 95% CI 0.07–0.85, p=0.003). Patients with MRSA and MSSA were similar in all other respects evaluated.

Our data are not sufficient to detect clinically important outcome differences between MRSA and MSSA among patients with SAP, possibly due to the small number of subjects (Table 4). Forty-two percent of MRSA patients compared to 20% of MSSA patients were admitted to the ICU (OR 2.9, 95% CI 0.7–12.6, p=0.15). Patients with MRSA had a median length of hospital stay of 19 days compared to 12 days for those with MSSA (OR 1.04 per additional day, 95% 0.99–1.1, p=0.15). MRSA patients experienced a combined outcome of ICU admission, intubation, and/or chest tube 58% of the time, compared to 30% of the time for MSSA patients (OR 3.2, 95% CI 0.9–11.3, p=0.07). In addition, no differences were found between MRSA and MSSA patients in death, risk for intubation, or length of ICU stay.

## Discussion

This case-control study compares two groups of hospitalized, HIV-infected patients with a well-documented etiology of pneumonia – *Staphylococcus aureus* or *Streptococcus pneumoniae* – and finds that patients with SAP experience longer hospital courses and more morbidity than those with SPP. Patients with SAP have more co-morbidities, more severe disease and more complicated hospital courses, and are more likely to be readmitted within 2 months. The role drug resistance in *S. aureus* plays is unclear, though it remains possible that MRSA is contributing to the excess morbidity of SAP.

The notable differences in the populations – more co-morbid conditions and possibly a higher prevalence of chronic lung disease – suggest that differences in transfer to ICU, respiratory failure, or length of stay may be partly explained by pre-existing conditions. Previous studies have documented the association between co-morbid conditions, risk for acquiring SPP and SAP, and poor outcomes [8]. Cirrhosis is a predictor of CAP-related mortality in people with HIV, and Manno et al. showed that cirrhosis increases the odds for death and significantly prolongs hospital stay [7, 9]. Cirrhosis has also been identified as a risk factor specifically for SAP in patients with HIV [13]. In our study, 73 patients had hepatitis without documented cirrhosis and an additional 8 had cirrhosis. While we did not demonstrate a mortality difference due to SAP, the preponderance of liver disease among SAP cases may be driving the number of ICU admissions and prolonged hospital stays.

The difference in pleural space complications seen in this study is also consistent with existing knowledge of *S. aureus*- related infections and may worsen outcomes. Tumbarello et al. report pleural effusion in 31% of 24 episodes of SAP, including one pleural space *S. aureus* infection and one lung abscess infected with *S. aureus* where involvement of the pleura is unclear [13]. The ability of recently emergent strains of community-acquired MRSA (USA300) to cause tissue necrosis is well documented, primarily in studies of skin and soft tissue infections [19], though studies of pneumonia in immune competent populations are emerging [20–22]. We were unable to document lung necrosis in this study, but the prevalence of pleural space infection was high (12% with pleural fluid cultures positive for *S. aureus*). MRSA may have contributed to the prevalence of this finding: of 8 SAP patients requiring a chest tube, 6 had MRSA; of 15 treated in the ICU, 11 were MRSA; and of 12 intubated, 8 were MRSA. These findings are consistent with MRSA-related trends in our data but are not conclusive. The study numbers may have been too small to detect true differences. The MRSA-related findings – odds of chest tube 2.7 (p=0.26), of intubation 1.8 (p=0.45), and of ICU 2.9 (p=0.15); median increase of 7 days in the hospital (p=0.15) – may warrant further investigation in a larger study.

The strengths of this study include the large number of SAP cases, which allowed for extensive comparisons between SAP and SPP, and a strict microbiological definition of pneumonia type, which allowed for clear comparisons between etiologies and a better assessment of the impact of microbiology, as opposed to HIV status, on outcome. The study has limitations as well. Retrospective studies must rely on limited clinical data and administrative classification of disease that might not adequately capture differences between patient populations. Pneumonia is a clinical diagnosis without a simple, reliable diagnostic test, making disease classification for research purposes inherently fraught; this study emphasized microbiological characterization, as noted, but likely selected a somewhat sicker population of both SAP and SPP as a result. We also did not systematically collect data to provide a severity of illness score, such as a Pneumonia Severity Index (PSI). However, our patients are well characterized in terms of the severity of their clinical illness. Vital status at 6 weeks following discharge could not be confirmed in 4 patients – 2 with SAP and 2 with SPP – raising the potential for a biased estimate of mortality difference between groups, though this seems unlikely. Despite the relatively large sample size and the long study period, the study may still have been underpowered, in particular with respect to MRSA.

Patients with HIV diagnosed with pneumonia due to *S. aureus* were sicker at baseline, had more recent contact with the healthcare system, and experienced longer, more complicated hospitalizations. Pneumonia due to MRSA warrants further study as a contributor to the excess morbidity associated with *S. aureus* pneumonia in people with HIV.

## Figures and Tables

**Table 1:** Baseline characteristics of 130 HIV-infected patients with 147 pneumonia cases by type.

		*Staphylococcus aureus* N (%)	*Streptococcus pneumoniae* N (%)	p value
N		42	93	
Mean age ± SD, years		46 ± 9	44 ± 8	0.27
Male		32 (76)	68 (73)	0.71
Race	White/Caucasian	22 (52)	31 (33)	0.04
	Black/African American	19 (45)	54 (58)	0.17
	Other	1 (2)	8 (9)	0.27
Homeless		9 (21)	23 (25)	0.68
Current alcohol abuse (missing 1)		13 (31)	34 (37)	0.50
Current IDU (missing 1)		7 (17)	26 (28)	0.15
Current tobacco use (missing 1)		21 (50)	74 (80)	<0.001
Median CD4µL cell count (IQR), cells per		185 (76–386)	239 (94–442)	0.27
Geometric mean HIV viral load (95%CI), copies per mL		4750 (1658–13,610)	12,238 (6834–21,913)	0.09
Median WBC count (IQR), cells×10^3^/mm^3^		9.3 (5.7–13.3)	10.7 (7.7–16.6)	0.09
Mean O_2_ saturation§±SD		91 ± 6	92 ± 5	0.46
Prior pneumococcal vaccination (missing 3)		7 (18)	21 (23)	0.49
PCP prophylaxis		23 (55)	20 (22)	<0.001
TMP-SMX to prevent PCP		14 (33)	14 (15)	0.02
Chronic lung disease		20 (48)	28 (30)	0.05
Other co-morbid diagnosis*		37 (88)	60 (65)	0.004

Note: IDU=intravenous drug use; PCP = *Pneumocystis jirovecii* pneumonia; WBC = White Blood Cell; TMP-SMX= Trimethoprim-Sulfamethoxazole; SD= Standard Deviation, IQR= Inter-Quartile Range, CI= Confidence Interval

§ O2 saturation indicates percent hemoglobin oxygen saturation breathing ambient air

* Co-morbid diagnoses include any one or combination of the following: hepatitis without cirrhosis (n=77), hepatitis with cirrhosis (n=8), anemia (n=10), chronic renal insufficiency (n=5), diabetes mellitus (n=4), cancer (n=4), congestive heart failure (n=3), coronary artery disease (n=2), Immune Thrombocytopenic Purpura (ITP) (n=2), Thrombotic Thrombocytopenic Purpura (TTP) (n=1), Kaposi’s malignancy (n=2), pancytopenia (n=1), protein C&S deficiency (n=1), history of splenectomy (n=1), stroke (n=1)

**Table 2:** Predictors of *Staphylococcus aureus* versus *Streptococcus pneumoniae* pneumonia among people with HIV in 147 episodes.

	Unadjusted OR (95% CI), p value	Adjusted OR (95% CI), p value
**Health history**		
Any antibiotics	4.7 (2.2–10.1), <0.001	3.5 (1.3–9.9), 0.02
Prophylactic antibiotics	5.1 (1.6–16.6), 0.01	Excluded*
Non-prophylactic antibiotics	2.3 (1.0–5.1), 0.04	Excluded*
PCP prophylactic antibiotics	3.9 (1.8–8.4), <0.001	Excluded*
Co-morbid diagnosis	4.5 (1.6–12.5), 0.003	4.2 (1.1–16.6), 0.04
Recent healthcare contact	9.7 (3.8–24.9), <0.001	12.0 (3.8–38.3), <0.001
**Labs at admission**		
CD4 count (missing 3) (odds per 20 cell increase)	0.98 (0.95–1.02), 0.29	1.03 (0.97–1.08), 0.33
Viral load (missing 9) (odds per 500 copies increase)	0.7 (0.5–1.04), 0.08	0.8 (0.46–1.33), 0.37
WBC count (odds per 1x103 cell increase)	0.95 (0.90–1.01), 0.08	0.94 (0.88–0.99), 0.03

Note: PCP = *Pneumocystis jirovecii* pneumonia; WBC = White Blood Cell

* Prophylactic antibiotics, non-prophylactic antibiotics, and PCP prophylactic antibiotics were highly correlated, and could not be analyzed in the multivariate model

**Table 3:** Outcomes in 147 episodes of pneumonia in people with HIV by pneumonia type.

	*Staphylococcus aureus* N (%)	*Staphylococcus pneumonia* N (%)	OR (95% CI), p value
N	47	100	
Chest tube	8 (17)	7 (7)	2.7 (0.9–8.2), 0.07
Intubated/Mechanical ventilation	13 (28)	11 (11)	3.1 (1.2–7.9), 0.02
Treated in ICU	16 (34)	16 (16)	2.7 (1.2–6.1), 0.02
Treated in ICU, intubated &/or	22 (47)	20 (20)	3.5 (1.6–7.5), 0.001
chest tube			
Median days in hospital (IQR)	15 (6–21)	5 (4–8)	1.08 (1.02–1.1), 0.01
Median days in ICU (IQR) (of 32)	6 (3–14)	4 (3–11)	1.01 (0.9–1.1), 0.81
Readmitted (of 143)	22 (49)	21 (21)	3.5 (1.6–7.5), 0.001
Died during hospital stay	2 (4)	2 (2)	2.2 (0.3–15.7), 0.44
Died within 6 weeks after discharge (of 143)	3 (7)	2 (2)	3.4 (0.6–21), 0.18

Note: ICU = Intensive Care Unit; IQR = Inter-Quartile Range

**Table 4:** Outcomes of 46 episodes of *Staphylococcus aureus* pneumonia by susceptibility.

Outcomes	Total N (%)	Methicillin Resistant N (%)	Methicillin Susceptible N (%)	OR (95% CI), p value
N (% of total)	46 (100)	26(57)	20 (43)	
Chest tube	8 (17)	6 (23)	2 (10)	2.70 (0.5–15), 0.26
Intubated	12(26)	8 (31)	4 (20)	1.78 (0.4–8.0), 0.45
Treated in ICU	15(33)	11(42)	4 (20)	2.9 (0.7–13), 0.15
ICU, intubation or chest tube	21(46)	15(58)	6 (30)	3.2 (0.9–11), 0.07
Median days in ICU (IQR) (of 15)	5 (1–21)	3 (1–21)	8 (3–19)	0.98 (0.85–1.13), 0.73
Median days in hospital (IQR)	14 (6–21)	19 (9–23)	12 (5–17)	1.04 (0.99–1.1), 0.15
Readmitted (of 44)	21(48)	13(52)	8 (42)	1.5 (0.4–5.4), 0.54
Died during admission	2(4)	1(4)	1 (5)	0.8 (0.05–12). 0.85
Died at 6 weeks after discharge (of 44)	3(7)	2(8)	1 (5)	1.7 (0.2–19), 0.66

Note: MRSA = Methicillin-resistant *Staphylococcus aureus*; MSSA = Methicillin-suscep *Staphylococcus aureus*; ICU = Intensive Care Unit; SD = Standard Deviation
